# Collaborative knowledge mobilisation to increase awareness and use of new SNOMED clinical terms for fetal alcohol spectrum disorder in England

**DOI:** 10.3389/fpubh.2026.1794809

**Published:** 2026-08-31

**Authors:** James T. I. Parsonage, Amy Dillon, Louise Millington, Raja A. S. Mukherjee, Sandra Ionno Butcher, Andy Boyd, Patricia D. Jackson, Cheryl McQuire

**Affiliations:** 1 Bristol Medical School, University of Bristol, Bristol, United Kingdom; 2 School for Public Health Research (SPHR), National Institute for Health and Care Research (NIHR), Newcastle, United Kingdom; 3 NHS Bristol, North Somerset and South Gloucestershire Integrated Care Board (BNSSG ICB), Bristol, United Kingdom; 4 NIHR Bristol Biomedical Research Centre, University of Bristol, Bristol, United Kingdom; 5 South Yorkshire Integrated Care Board (ICB), Sheffield, United Kingdom; 6 Surrey and Borders Partnership NHS Foundation Trust, Surrey, United Kingdom; 7 University of Surrey, School of Medicine, Surrey, United Kingdom; 8 National Organisation for FASD, Hertfordshire, United Kingdom; 9 Past President and Continuing Member of Scottish Paediatric Society, Past Chair and Continuing Member Scottish Association of Community Child Health, London, United Kingdom; 10 Paediatric Representative Scottish Health Action on Alcohol Problems, Sponsored by Royal College of Physicians of Edinburgh, Edinburgh, United Kingdom

**Keywords:** clinical coding, electronic patient record (EPR), fetal alcohol spectrum disorder (FASD), knowledge mobilisation, primary care/general practice < setting of care

## Abstract

**Introduction:**

Fetal alcohol spectrum disorder (FASD) affects an estimated 1.8–3.6% of UK school children and up to 27% of children in care. FASD remains vastly under-diagnosed and under-recorded in UK electronic patient records (EPRs), leading to significant challenges for individuals with the condition. In 2024, we developed new SNOMED clinical terms aligned to UK national guidelines and quality standards for FASD.

**Aims:**

Through collaborative knowledge mobilisation (KM), we aimed to: i) raise awareness of the importance of utilising these new clinical terms; ii) identify barriers to FASD code usage in primary care EPRs; iii) co-develop creative communication materials and dissemination strategies.

**Methods:**

We engaged with stakeholders (*N* = 18) including paediatricians, general practitioners, wider health professionals, data specialists, third-sector representatives, and researchers, through a workshop and individual meetings. Individuals living with FASD provided their perspectives on the importance of recognising FASD in EPRs, for inclusion in communication materials.

**Results:**

We co-produced free, publicly available creative communication materials including an animation, infographics and social media images. Materials focus on raising awareness of new SNOMED clinical terms for FASD, address stakeholder reported barriers to clinical coding, and provide actionable recommendations to improve clinical coding for FASD within primary care electronic records, and diagnostic services. We promoted these materials through social media platforms, a press release, conference presentations, and an editorial.

**Discussion:**

Our collaborative KM work provides an exemplar for developing materials with the aim of supporting improved clinical coding in EPRs. Further work is required to maximise and evaluate the impact of our materials.

## Introduction

Fetal alcohol spectrum disorder (FASD) affects an estimated 1.8–3.6% of UK school children and up to 27% of Children in Care (1, 2). It is caused by prenatal alcohol exposure (PAE) and characterised by a range of neurodevelopmental impairments in learning, memory, attention, social and emotional regulation. Less than 10% of individuals with FASD have co-existing dysmorphic facial features (called sentinel facial features) which include a smooth philtrum, thin upper lip and short palpebral fissures (eye openings) (3). More commonly, FASD presents with no dysmorphic facial features (3). Often early in life, neurodevelopmental deficits may appear mild, their severity growing or becoming more apparent as the need to employ more complex thinking skills grows (4). At presentation, such individuals may not meet a formal FASD diagnosis threshold and may be designated as ‘at-risk of neurodevelopmental disorder or FASD’ with a requirement for subsequent follow-up and re-assessment (4).

Clinical professionals describe a lack of confidence in recognising and managing the condition and report worries around potential stigma surrounding FASD diagnosis (5). Less than a quarter of general practitioners (GPs) report receiving training in asking mothers about alcohol consumption during pregnancy (5). Initiating and sensitively handling conversations around alcohol use during pregnancy and later at childhood presentation of possible FASD enhances reporting accuracy (6). It is also crucial for building trust between mothers and medical professionals, addressing stigma, reducing prenatal alcohol intake and making an FASD diagnosis (6).

In England, Primary Care Teams (PCTs) in General Practice (Family Medicine), like all NHS health and care systems, must use the machine-readable structured vocabulary entitled Systematised Nomenclature of Medicine—Clinical Terms (SNOMED CT) to record clinical information and diagnoses (7). Internationally, the not-for-profit organisation SNOMED International (8) determines global standards for health terms within its structured vocabulary and creates release versions that are, to an extent, locally adaptable by its member countries. Since 2016, the English version, entitled SNOMED CT UK Edition [currently distributed in Release Format 2 (RF2)], has conformed to the information standard ISN SCCI0034, (9, 10) and is technically supported by NHS England. SNOMED CT is expected to be soon introduced throughout Scotland and Wales. The emergence of SNOMED CT has yielded numerous advantages over previous systems (e.g., “Read code” systems, still currently in place in Scotland), which in practice promoted diversity and inconsistency in clinical coding (11). SNOMED CT ensures a common, hierarchical clinical language in electronic patient records (EPRs), reducing the potential for ambiguous or often misinterpreted terminology between healthcare teams (7). Further, SNOMED CT enables interoperability (easier clinical data transfer) between current clinical systems whilst ‘future-proofing’ data transfer for future clinical systems (7).

In 2019, the Scottish Intercollegiate Guidelines Network (SIGN) 156 guidelines, sought to unify UK FASD diagnostic terminology with the Canadian FASD Research Network (CanFASD) guidelines (4, 12). However, as specialist diagnostic language changed, English GP clinical recording systems (such as EMIS and SystmOne), did not contain SNOMED CT terms which matched the latest diagnostic terminology. This absence of clinical codes for the full FASD spectrum has contributed to an under-recording of FASD in EPRs (1, 13, 14).

It is important to note that SNOMED CT is a reference terminology for representing clinical content in EPRs, rather than a diagnostic instrument or classification. Diagnostic decisions sit upstream of terminology selection and should be made and documented using relevant clinical criteria and guidance, including SIGN 156 (4), the National Institute for Health and Care Excellence Quality Standard for FASD (NICE QS204) (15) and, where applicable, ICD-11 diagnostic criteria (16). As a coding and reporting instrument, the International Classification of Diseases (ICD), maintained by the World Health Organization (WHO), provides the international classification framework for morbidity and mortality reporting. Relevant ICD categories for FASD include, for example, ICD-11: LD2F.00 Fetal alcohol syndrome; 6A0Y Other specified neurodevelopmental disorders, to which the entity “neurodevelopmental syndrome due to prenatal alcohol exposure” is indexed; KA06.2 Fetus or newborn affected by maternal use of alcohol; and ICD-10 (17): Q86.0 Fetal alcohol syndrome, dysmorphic; appropriate codes in blocks F80-F89 Disorders of psychological development and F90-F98 Behavioural and emotional disorders with onset usually occurring in childhood and adolescence; and P04.3 Fetus and newborn affected by maternal use of alcohol. SNOMED CT-coded primary care data may therefore support reporting, audit, surveillance and research, but fully interoperable use requires explicit mapping and aggregation to relevant ICD-10 or ICD-11 categories. In England, mapping from SNOMED CT UK Edition (source) to ICD 10 is supported and documented by NHS England’s terminology services (18).

Under-diagnosis and under-recording of FASD reflect distinct but sequential challenges within the clinical information pipeline. Clinical recognition and formal diagnosis typically occur in specialist services, after which the diagnosis must be clearly documented in correspondence to primary care, such as clinic letters or discharge summaries. Only then can primary care teams enter a structured diagnostic code in the EPR using SNOMED CT; subsequent aggregation of coded data for audit, surveillance and research represents a further step.

The focus of this project is not on addressing all stages of this pathway, nor on resolving the wider problem of FASD under-diagnosis. Rather, it focuses on improving awareness and use of appropriate SNOMED CT terms in English primary care records, and on supporting clearer alignment between specialist diagnostic terminology and primary care coding. Improved clinical coding is therefore an important component of better FASD recording and surveillance, but remains conditional on upstream clinical recognition, diagnosis and documentation.

As of October 2025, approximately 64 million patients were registered in English General Practice (19). With SNOMED CT as the universal coding language, as mandated by NHS England, in theory, there should be ample data for the understanding of patterns of health and outcomes for those with FASD. However, there were only 1,100 uses of active diagnoses [fetal alcohol syndrome (FAS) or fetal alcohol spectrum disorder] in English primary care between August 2020 and July 2021 (20). Considering conservative estimates of a 1.8% prevalence of FASD, when applied to a population of roughly 9 million pupils attending school in England, this gives a crude expected prevalence of 162,000 affected pupils (1, 21). Under-recognition in healthcare data has contributed to a scarcity of research and evidence-based support to improve care for people with FASD. Without FASD diagnoses recorded in EPRs, those with the condition often find themselves unable to access proportionate physical, mental health and social support.

The prior terms in SNOMED CT that were available for use in primary care records did not represent the diagnostic terminology recommended by the current NICE and SIGN guidelines and used by specialists (4, 15). Further no term in SNOMED CT existed for those ‘At increased risk for fetal alcohol spectrum disorder’, which would enable identification of those with milder neurodevelopmental deficits which later may become more severe, for whom follow-up and/or reassessment is recommended (4).

In April 2024, members of the project team (PJ, CM, AD) led a successful application via NHS England for new SNOMED CT UK Edition (22) clinical terms to represent the full spectrum of FASD, aligned with NICE and SIGN guidance (4, 15). Implementation of these terms in English primary care systems may support more accurate and consistent recording of FASD in electronic patient records (EPRs), where clinical recognition, diagnosis, and documentation have already occurred. Improved recording may, in turn, strengthen care planning, audit, surveillance, and monitoring of progress against the NICE quality standard for FASD, including standards relating to prenatal alcohol exposure assessment, referral for neurobehavioural assessment, and management planning for people living with FASD (15).

The newly introduced SNOMED CT UK Edition terms are:

1) Fetal alcohol spectrum disorder with sentinel facial features [disorder] SNOMED CT code: 1894471000000108.
2) Fetal alcohol spectrum disorder without sentinel facial features [disorder] SNOMED CT code: 1894461000000101.
3) At increased risk for fetal alcohol spectrum disorder [finding] SNOMED CT code: 2078801000000102.

Now implemented across English primary care clinical recording systems (both EMIS and SystmOne), successful knowledge mobilisation (KM) is crucial for raising awareness and promoting uptake. Our team secured funding for collaborative KM to achieve three key aims, to:

- Share knowledge about the availability/importance of utilising FASD clinical coded terms in EPRs.
- Discuss and address facilitators/barriers to FASD coding within primary care EPRs.
- Co-develop, with stakeholders, “creative communication”/KM materials and dissemination strategies.

Creative communication involves the production of KM materials to share information in ways that move beyond traditional academic formats. In our case, this included an animation, infographics and social media thumbnails (23). Here, we describe our approach to collaborative KM to develop KM materials to meet our three aims.

## Methods

Our project team consisted of academic researchers, clinicians, third-sector representatives, data governance and clinical terminology specialists. We incorporated National Institute of Health and Care Research (NIHR) guidance on planning KM (24).

**Figure 1:**
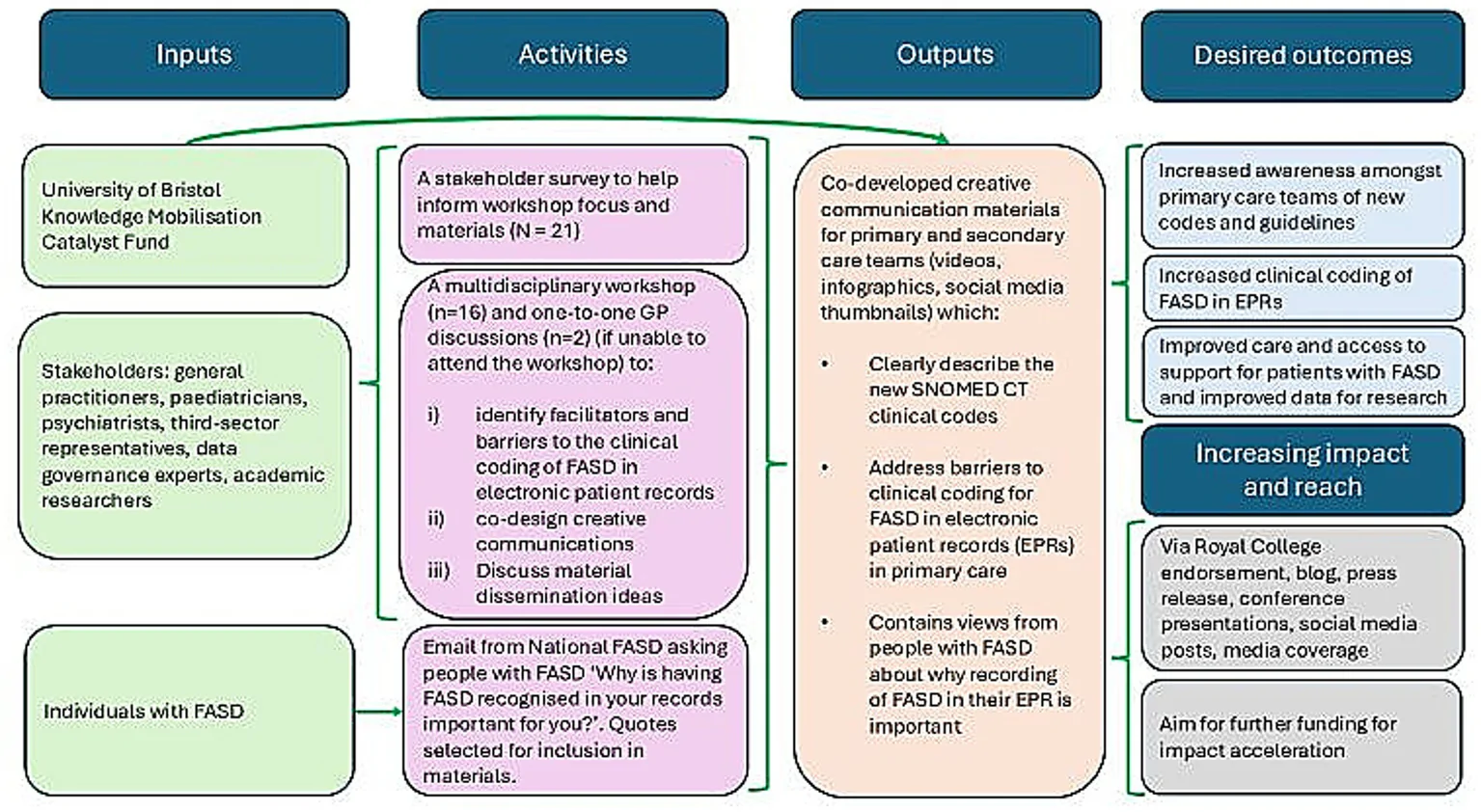
Logic model of collaborative knowledge mobilisation.

**Figure 2:**
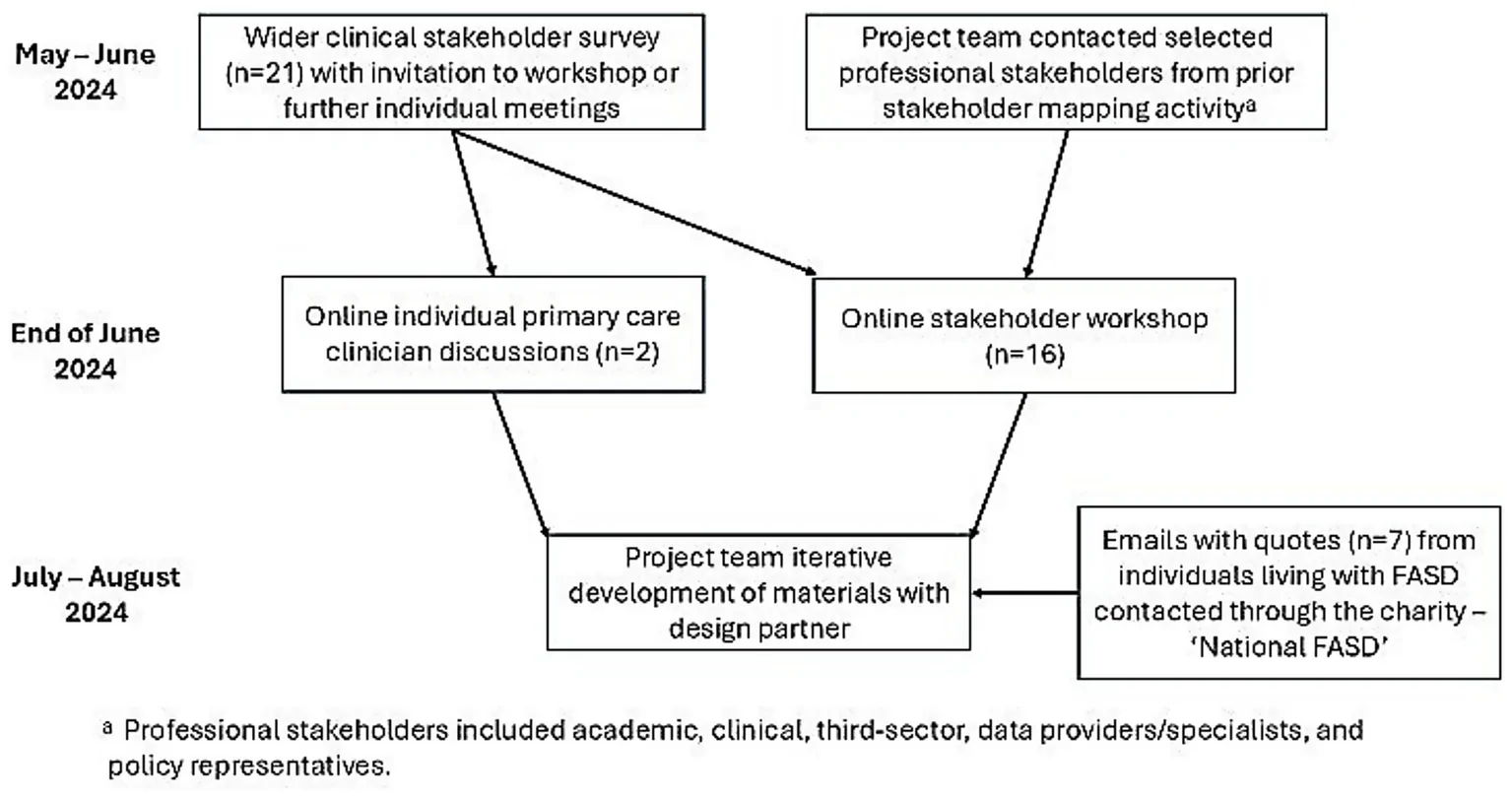
Flow diagram of stakeholder engagement and public involvement activities.

Figure 1 presents the logic model and Figure 2 is a flow diagram of our stakeholder consultation activities.

The simple logic model describes how, after establishing funding for our KM materials, we envisaged that the combination of stakeholder co-development workshops and public involvement of individuals with FASD, would lead to the production of materials that met our key aims. The desired outcomes of these materials are that they will improve awareness of both the new FASD coded terms and the latest guidelines for care, together with increased clinical coding of FASD in primary care records, translating to improved care for individuals with the condition. Below, we describe the process of the co-development of our KM materials and initial dissemination.

Formal ethical approval was not required as our project consisted of patient and public involvement (PPI) and stakeholder engagement activities, rather than research (25). We, however, took steps to ensure a collaborative atmosphere with stakeholders. For example, we recognised that busy clinical environments are often non-conducive to accurate and consistent clinical coding, taking care that discussions of barriers to clinical coding were focused on collective learning, not critique of practitioners’ coding experiences. We also involved individuals with FASD via a trusted third-sector organisation – the National Organisation for FASD (National FASD).

Our KM work followed these steps: i) stakeholder identification and prioritisation; ii) a stakeholder survey to inform workshop materials (*n* = 21); iii) a multi-disciplinary stakeholder workshop (*n* = 16) and individual discussions with GPs (*n* = 2); iv) email contact, facilitated by the National FASD, to obtain quotes from people with FASD explaining the importance of having their condition recorded; v) iterative creative communication material creation; vi) dissemination; vii) measuring reach and feedback. These steps are described in more detail below.

## Stakeholder consultation

### Stakeholder identification and prioritisation

A comprehensive stakeholder mapping exercise was performed by CM and AD as part of a previous project to understand the acceptability and feasibility of a national linked database for FASD (26). This provided a range of professional stakeholders and organisations (third sector, clinical, academic, data providers/specialists, and policy representatives) who were contacted via email for involvement in our workshop. We prioritised inviting a mixture of primary care and diagnosing clinicians.

### Stakeholder survey

We used a Microsoft Forms survey (see Supplementary material S1) to gather information on barriers to clinical coding of FASD (27). This information informed topic guide development for our workshop around barriers to the clinical coding of FASD. Respondents could also express their interest in attending our workshop via the survey. We also gathered information about respondents’ regional location, clinical specialty, confidence and usual method of clinically coding for FASD and an indication of favourability for the new terms. The survey was shared with a weblink and QR code on X/Twitter and the Future NHS collaboration platform. We asked contacts from the Greater Manchester Integrated Care Board, Bristol, North Somerset and South Gloucestershire One Care Consortium, Bristol’s Drug and Alcohol Health Integration Team, and the University of Bristol’s Centre for Academic Primary Care, Centre for Academic Child Health, and Centre for Academic Mental Health to share the survey with clinical colleagues.

We received *n* = 21 responses from GPs (*n* = 11), paediatricians (*n* = 8), a nurse and a psychotherapist. We are unable to give an exact response rate for our survey given the range of channels that we used to publicise it (described above).

### Multidisciplinary stakeholder workshop and individual in-depth discussions with GPs

We invited healthcare contacts who had responded to our survey or emails to participate in a one-hour workshop. The topic guide (see Supplementary material S2) included discussion prompts on:

1) How we can effectively share knowledge about up-to-date, accurate and consistent clinical coding of FASD in clinical systems, with primary and secondary care teams (including diagnosing clinicians).
2) How to address the practical considerations, facilitators and barriers to recording a diagnosis of FASD in EPRs.
3) The best format and content for creative communication materials to support effective KM of FASD clinical coded terms.

The workshop was recorded via Microsoft Teams with the consent of all participants (*n* = 16), to support notetaking. We also held 1-h online individual meetings (*n* = 2) with GPs who could not attend the workshop.

### Emails to people with FASD

Workshop responses emphasised that reflecting how FASD clinical coding was important to those with FASD in our KM materials, was critical. National FASD, emailed adults and young people in their FASD Advisory Committee and included a post on their Facebook social media page, which asked “Can you please give a quote about why you think it’s important to have FASD noted in your NHS records? Do you want doctors and others caring for you to know you have FASD? Why does that help?”. Seven people with FASD responded.

## Development and dissemination

### Development of creative communication materials

In collaboration with a graphic design partner, we devised a script and aesthetic for the KM materials based on information gathered through the stakeholder consultation. The project team chose 2 quotes from the seven respondents with FASD to include in our KM materials, based on their clarity, conciseness and relevance to PCTs. Iterative script development with our graphic design partner occurred via emails and team meetings until members of the project team reached agreement that the outputs had achieved our KM aims.

### Dissemination of outputs

Our KM materials received endorsement from the Royal College of Psychiatrists (RCPsych), and a statement of support from the Royal College of General Practitioners (RCGP). To reach GPs, a member of the project team (LM) presented our work at the RCGP’s annual conference and produced a video recording explaining the new SNOMED clinical terms and the rationale for their use, which was distributed together with our KM materials to English ICBs, national safeguarding teams and Named GPs (28, 29).

We published an associated editorial in the British Journal of General Practice with general recommendations for PCTs to support the prevention, diagnosis, clinical coding and management of FASD (30). Following a press release, the Pulse (a general practice news publication) featured our KM work (31). National FASD have included the new SNOMED clinical terms in their latest report entitled ‘Not Commissioned: Systemic confusion in NHS services for alcohol, pregnancy and FASD’ (32). CM, AD and SB presented our KM project as part of the Office for National Statistics (ONS) Research Excellence Series 2024 (33).

### Measuring reach and feedback

While not a formal component of the project, we collected *post hoc* information on the reach, acceptability, and use of the KM materials through online metrics (including views of the animation on YouTube), a dissemination log maintained by the study team (capturing conference presentations, blogs, and related activities), and feedback from third-sector and practice partners. This feedback was collected on an informal, *ad hoc* basis.

## Results

### Stakeholder survey

Responses from our survey indicated that barriers to clinical coding of FASD in EPRs that should be further discussed at our workshop included i) insufficient awareness about FASD and the latest guidelines; ii) concerns about stigma from coding FASD in EPRs; iii) the need for secondary care diagnosis prior to coding in primary care; iv) the need for FASD diagnostic terminology from secondary care diagnostic reports/letters to align with SNOMED clinical terms in primary care.

Stakeholders also pointed to difficulties in initiating conversations about, and recording, prenatal alcohol exposure (PAE), the causative agent of FASD. However, addressing this was considered outside of project scope, which focused specifically on addressing the use of FASD terms and codes within SNOMED CT based clinical records.

### Stakeholder workshop

Workshop participants suggested that KM materials must clearly signpost to the SIGN guidelines and NICE quality standards, given the lack of awareness of them in PCTs, and clearly describe the three new SNOMED clinical terms. There was agreement that having distinct diagnostic terms of FASD with and without sentinel facial features, would support future research and audit. Stakeholders felt that addressing language consistency between primary and secondary care was pivotal. They felt that supporting GPs with clear coding instructions through clinical letters from secondary care, should be a central feature of the KM materials.

GPs in individual discussions echoed this that they would not be comfortable with coding for FASD without explicit direction or diagnosis in clinic letters from secondary care. This was in part due to a feeling of not knowing what conversations regarding diagnosis had taken place within the families and variable understanding of the condition. Workshop attending secondary care clinicians however described that, by the stage of giving a diagnosis of FASD, such family/carer discussions would have already taken place in secondary care. GPs also felt that the closer diagnoses written clinic letters matched the new SNOMED clinical terms, the more likely those specific terms would be chosen.

During the workshop and individual GP discussions, there was agreement that materials must take a positive tone, encouraging greater recognition of FASD in clinical records to ensure better continuity of care. Including patient quotes from those with FASD was felt to be essential for representing the perceived positive impacts of coding for FASD on their care.

There was consensus that the primary audience for our produced materials were primary care doctors, however this was refined through individual discussions with GPs, to the wider primary care team (PCT). Primary care doctors informed us that primary care clinical coding is undertaken by a range of primary care staff, including administrative and non-clinical staff.

The most requested forms of creative communication by clinicians were a short video and infographic, as due to busy clinical work demands, these formats would enable rapid and accessible knowledge transfer. For successful dissemination of materials, participants felt that materials must be deemed trustworthy and aligned with organisations representing clinicians and those with FASD. Primary and secondary care clinicians suggested several options for disseminating the outputs. This included seeking Royal College endorsement for the materials, inclusion at training days, or presentation at GP focused conferences, and sharing through Integrated Care Boards (ICBs) or general practice newsletters.

### How our materials addressed barriers to the clinical coding of FASD

The project team aimed to address key barriers identified through the survey and workshop related to: i) coding FASD in EPRs; and ii) accessing and using our KM materials. Table 1 summarises these barriers and shows how they informed the design of our materials, thereby enhancing the reproducibility of our approach. We draw on this table below to describe the key features of the materials.

**Table 1:** Barriers for clinical coding of FASD for primary care teams and how they influenced our KM materials to support the clinical coding of FASD.

Barrier type	Barrier identified	How did this influence KM material design?
Barriers to clinical coding of FASD in electronic patient records	Insufficient awareness of latest FASD guidelines in primary care teams.	In the materials we signpost immediately to the latest SIGN guidance and NICE quality standards and include a direct link to the SIGN guidance via a QR code.
	Concerns about stigma from coding for FASD.	We focused on the benefit that can be achieved through coding the condition in primary care EPRs. Using quotes from individuals with FASD we emphasise the importance of recognition of FASD to individuals with the condition.
	The need for secondary care diagnosis prior to coding in primary care.	Our materials address both secondary care and primary care teams, emphasising the role of secondary care teams to support the clinical coding of FASD in primary care. This includes emphasising the need for clinic letter terminology to match the new diagnostic SNOMED clinical terms in primary care to facilitate coding.
	The need for FASD diagnostic terminology from secondary care diagnostic reports/letters to align with SNOMED clinical terms in primary care.	
	General practitioners described that a wide range of primary care team staff engage in clinical coding, who may share lack of awareness around the latest FASD guidelines.	To ensure that materials were not solely focused on general practitioners, materials addressed the wider primary care team (PCTs) who may be involved in clinical coding.
Barriers to accessing and utilising materials	Busy clinical workloads leave limited time to access learning information.	Materials were designed to be short and give the important information succinctly. The video was 1 min long, and the infographic was a single page.
	Primary care teams and health organisations may want to utilise and embed the materials in their practices or webpages.	All materials were made freely available via Open Science Framework to any individual to use them as needed, such as embedding them in local websites or information for healthcare professionals.
	Primary care teams felt that materials should carry official endorsement from Royal College to ensure they could be seen as trusted.	We received official endorsement from the Royal College of Psychiatrists (shown in the materials) and a statement of support from Royal College of General Practitioners.

**Figure 3:**
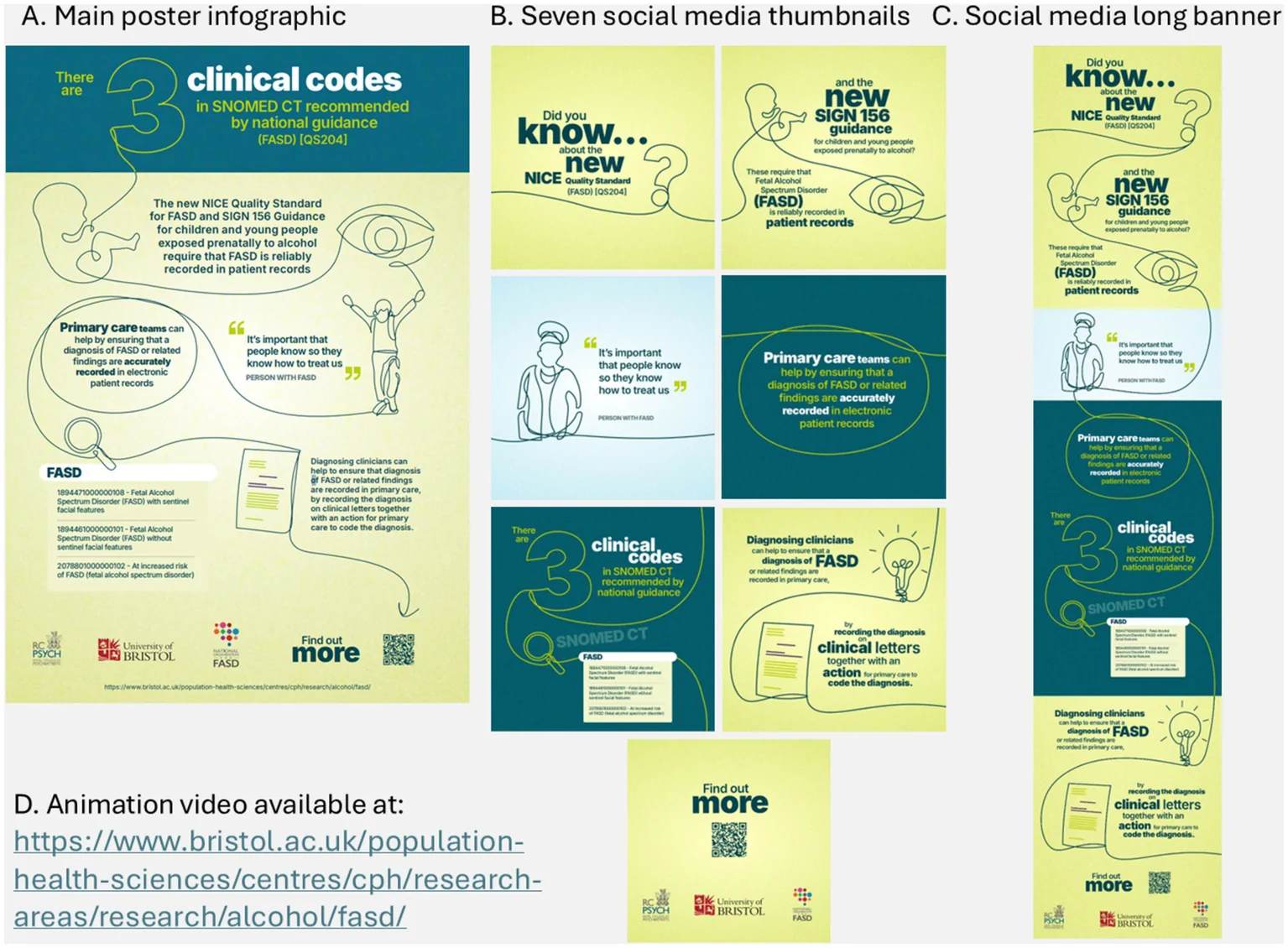
Image showing our main infographic **(A)**, social media thumbnails **(B)**, social media long banner **(C)** and the link to our webpage containing our video animation **(D)**.

Figure 3A presents the main poster infographic. To support the adoption of updated terminology, whilst reinforcing the importance of coding FASD in primary care, the poster prominently displays three SNOMED clinical terms within a coding search box, reflecting how clinicians interact with IT systems such as EMIS.

In response to limited awareness of the latest SIGN guidelines and NICE quality standard for FASD, and the need for consistent recording in clinical records, these guidelines are clearly highlighted. The materials also include a QR code linking directly to the SIGN 156 guideline on children and young people prenatally exposed to alcohol, enabling easy access for clinicians.

As described, clinicians felt that stigma surrounding the recording of FASD could be reduced by clearly emphasising the importance of documenting the condition for affected individuals. This importance is illustrated with a positive quote from a patient with FASD. To further support PCTs, guidance is provided on how diagnosing secondary care clinicians (paediatricians or psychiatrists) can assist by explicitly stating the diagnosis in clinical letters. This reflects the need for clear communication from secondary care teams to enable accurate clinical coding of the condition.

We also produced a 1-min short animation (Figure 3D), containing information about NICE and SIGN guidance for FASD, the new SNOMED clinical terms, quotes from people living with FASD and links to our project website with a QR code linking to our University of Bristol webpage. The QR code destination difference was intentional, with the design team feeling that the infographic would be more likely to be displayed in primary care settings, where guideline access would need to be faster for patient care, whereas the video would benefit from direction to our website with more information around the project itself with additional links to relevant guidelines. The animation follows the same design language and addresses the same barriers to clinical coding of FASD as our infographic.

The brevity of the animation, complements the single page infographic, addressing the barrier expressed by GPs that the materials needed to convey information rapidly, given their extensive workload. Clinicians emphasised that endorsement from a recognised professional body would be important for boosting the credibility and trustworthiness of the materials. In response, we sought and obtained endorsement from the Royal College of Psychiatrists, and its logo was included on all materials to signal this endorsement.

Seven social media thumbnails (Figure 3B) were produced based on the distinct frames of the animation together with a final combined social media long banner which included all of the distinct animation frames (Figure 3C).

All materials are freely available via Open Science Framework (OSF) and are described in a University of Bristol webpage and blog (34–36). Making the materials freely available supports their integration within local health systems, including incorporation into local documentation and websites, as well as display within healthcare settings where appropriate.

### Measuring reach and feedback

Our YouTube animation has, at the time of writing (23rd March 2026) only had limited engagement with 282 views (37). Unfortunately, social media information on posts are unavailable as researchers have migrated from X/Twitter where they were originally posted. In addition, we do not have access to webpage traffic information. We are also unable to capture information around the number of downloads of our materials on OSF.

We are however aware that positive impacts have emerged from our work through responses from stakeholders. Following our material release, the three described FASD terms have also been added to the Royal College of Paediatrics and Child Health (RCPCH) paediatric core reference set. This will help ensure consistent FASD terminology across paediatric and primary care datasets.

We also received feedback from people living with FASD which was generally very positive. However, in practice, since implementation of the new codes, some FASD patients have reported confusion, as their GPs have been reluctant to change prior recorded diagnoses from secondary care using older terminology to the new SNOMED clinical terms.

## Discussion

This article has described how our collaborative knowledge mobilisation methods, through consultation with a range of stakeholders, led to the co-production of engaging and accessible materials to increase awareness of UK FASD guidance and new FASD SNOMED clinical terms. Our materials addressed key barriers, emerging out of our engagement activities, to FASD clinical coding in primary care, shown in Table 1, whilst taking a multidisciplinary approach in providing guidance for how secondary care teams and specialists may support this process. Our collaborative knowledge mobilisation methodology may be helpful to those wishing to improve EPR clinical coding for other health conditions.

As described, we used a survey to inform our understanding of barriers to the coding of FASD in primary care, for which the response rate was likely very low. This means that the identified barriers cannot be considered representative of each professional group included. As such wider barriers may exist which our materials have not addressed.

While the primary aims of this project were the co-development of materials and initial dissemination described, there is evidence that the new codes are being used, albeit, in small numbers. Between August 2024 to July 2025, “Fetal alcohol spectrum disorder with sentinel facial features,” “Fetal alcohol spectrum disorder without sentinel facial features,” and “At increased risk of fetal alcohol spectrum disorder” were coded 50, 90 and 20 times, respectively, (38). Over this recorded data period, our materials were being finalised followed by their initial dissemination. This highlights the need to pursue further funding to maximise and evaluate the impact of these KM materials, more robustly tracking engagement with our materials, longitudinally examining changes in primary care use of these new FASD codes and understanding the impacts of their use on patient care. We plan to pursue such funding in the near future.

The feedback from individuals with FASD that primary care teams were reluctant to change older diagnostic terms for FASD for newer coded terms, suggests that further work towards understanding and addressing these issues is needed. For clarity, older diagnostic terminology for FASD, are fully transferrable to the new SNOMED clinical terms. For example, fetal alcohol syndrome (FAS) is synonymous with the new term “fetal alcohol spectrum disorder (with sentinel facial features)” and other spectrum diagnoses such as partial fetal alcohol syndrome (pFAS) without dysmorphic facial features can be transferred to the “fetal alcohol spectrum disorder (without sentinel facial features)”.

It is important to note that our project focused on one step in the clinical information pipeline for FASD—namely improved clinical coding in electronic patient records. It is important to acknowledge that this is only one part of the clinical information pipeline, which could be conceptualised as i) under-diagnosis of FASD, leading to ii) poorly documented FASD diagnoses in specialist correspondence; leading to iii) incorrect or absent code assignment in the EPR leading to iv) absent or incorrect tabulation and aggregation of granular codes into higher-level groupings for reporting. Our project did not develop or validate SNOMED CT-to-ICD mapping or aggregation rules; future work will be needed to determine how the new UK SNOMED CT FASD concepts should be linked to ICD-10 and ICD-11 categories for routine reporting, surveillance and international comparison.

Accurate and consistent clinical recording of FASD in EPRs is just one part of ensuring that those at risk of, or diagnosed with FASD receive the recognition, care and support they need. Consistent with national guidance, improving clinical coding of FASD in routine care must be matched with improved enquiry and recording of prenatal alcohol exposure (PAE) at healthcare appointments (15).

In addition, unlike for other common neurodevelopmental disorders including autism and attention deficit hyperactivity disorder (ADHD), the majority of NHS Integrated Care Boards do not commission FASD diagnostic services (32). This is a systemic bottleneck, where diagnosis of the condition lags behind true population prevalence (30). The ability to enhance population level primary care data to facilitate, understand, and improve prevention and outcomes for those living with FASD requires not only increased awareness and action on recognising FASD diagnoses in electronic patient records, but also improved reporting of prenatal alcohol exposure, along with national development of accessible FASD diagnostic and support pathways.

## Data Availability

The original contributions presented in the study are included in the article/Supplementary material, further inquiries can be directed to the corresponding authors.
