# Supplementary material for: Collaborative knowledge mobilisation to increase awareness and use of new SNOMED clinical terms for fetal alcohol spectrum disorder in England
Source: Front Public Health. 2026 Aug 31;14:1794809. doi: 10.3389/fpubh.2026.1794809 (PMC13572132; doi:10.3389/fpubh.2026.1794809)
Supplement: Supplementary file 1 [file Image_1.pdf]

University of BRISTOL

Understanding diagnosis and clinical coding for fetal alcohol spectrum disorders (FASD)

- **This short 5-minute questionnaire is aimed at understanding the barriers and facilitators to clinical coding of fetal alcohol spectrum disorder FASD in practice.**
- **All questions are optional, so feel free to complete as much as you are able to or would like to.**
- Fetal alcohol spectrum disorder (FASD) is under-represented in UK electronic health records (e.g. GP and hospital records). This is an issue because routine health data are important for helping us understand the prevalence, needs and outcomes of FASD in the UK population.

- Anonymous responses from this form will be used by a multidisciplinary team led by the University of Bristol.
- Responses will be used to inform an online consultation with clinicians to co-design materials to improve awareness and diagnosis of FASD and associated clinical codes.
- **If you would like to be involved in an online qualitative workshop to develop educational materials for FASD coding, please let us know in section 3.**

Many thanks in advance for your important contribution to this project.
Please contact Dr Cheryl McQuire (cheryl.mcquire@bristol.ac.uk) if you would like to discuss this project further.

The project team are:
Dr Cheryl McQuire: Research Fellow, Centre for Public Health, University of Bristol (lead)
Dr Amy Dillon: Data Access Lead, University of Bristol (project co-lead)
James Parsonage: NIHR SPHR Pre-Doctoral Fellow, Bristol Medical School, Centre for Public Health
Dr Patricia D. Jackson: FRCPCH Honorary Fellow University of Edinburgh, Co-Chair SIGN Guideline 156 (FASD)
Prof Raja Mukherjee: NHS Surrey and Borders Partnership, National Clinic for FASD
Sandra Ionno Butcher: CEO, National Organisation for FASD
Louise Millington: NHS South Yorkshire
Andy Boyd: Director UK Longitudinal Linkage Collaboration, Bristol Medical School
Janice Watson: Public Health Scotland Terminology Services

Please answer the questions below:

1

What Region of the UK do you work in?

- [ ] East Midlands
- [ ] East of England
- [ ] North East
- [ ] North West
- [ ] Northern Ireland
- [ ] London
- [ ] Scotland
- [ ] South East
- [ ] South West
- [ ] Wales
- [ ] West Midlands
- [ ] Yorkshire and the Humber

2

What is your clinical specialty?

- [ ] General Practice
- [ ] Paediatrics
- [ ] Psychiatry
- [ ] Other

3

In your role, do you encounter children/people with confirmed or suspected (FASD)?

- ○ Yes
- ○ No
- ○ Unsure

4

How confident would you say that you are in diagnosing FASD?

- ◯ Extremely confident
- ◯ Somewhat confident
- ◯ Somewhat not confident
- ◯ Extremely not confident

5

Please explain your answer to Q4

6

How likely would you be to use a clinical code for FASD on a relevant patient's electronic health record (e.g. using SNOMED CT or another system)?

- ◯ Extremely confident
- ◯ Somewhat confident
- ◯ Neutral
- ◯ Somewhat not confident
- ◯ Not confident at all

7

Please explain your answer to the above question

8

How would you usually clinically code for a patient who has **confirmed** FASD (e.g. using SNOMED)?

9

How would you usually clinically code for a patient who presents with **suspected** FASD (e.g. using SNOMED)?

Please read the following information and answer the questions below:

New codes for FASD have been introduced in 2024. These reflect NICE Quality Standard and Scottish Intercollegiate Guidelines Networks (SIGN) guidelines for FASD:

- **1894471000000108: Fetal Alcohol Spectrum Disorder with sentinel facial features (disorder)**
- **1894461000000101: Fetal Alcohol Spectrum Disorder without sentinel facial features (disorder)**

A further code for those with prenatal alcohol exposure, who may be at risk of FASD is also due to be launched:

- **At increased risk of Fetal Alcohol Spectrum Disorder** (code expected in June 2024)

More information on clinical codes here: https://digital.nhs.uk/services/terminology-and-classifications/snomed-ct

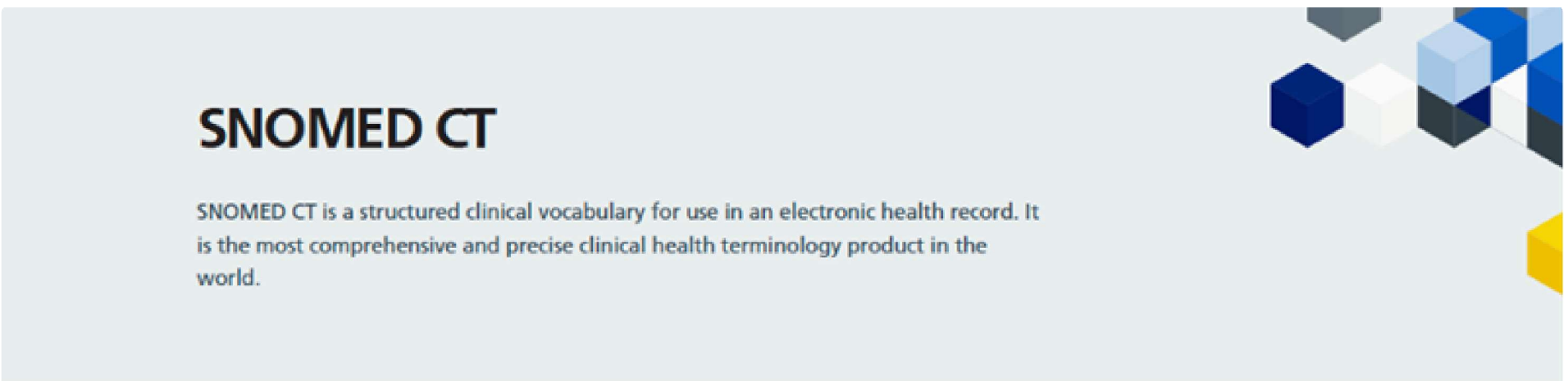

10

How likely would you be to use these new codes for FASD?

	Very likely	Somewhat likely	Neither likely nor unlikely	Somewhat unlikely	Very unlikely
Fetal Alcohol Spectrum Disorder with sentinel facial features (disorder)	○	○	○	○	○
Fetal Alcohol Spectrum Disorder without sentinel facial features (disorder)	○	○	○	○	○
At increased risk of Fetal Alcohol Spectrum Disorder	○	○	○	○	○

11

Given your responses to the above question, please explain your answers
(including any differences in your response for confirmed vs suspected FASD, where applicable)

12

Do you think that further educational content or communication materials around FASD diagnosis and clinical coding would be useful for clinicians

- ◯ Yes
- ◯ No
- ◯ Maybe

13

Are there any themes or learning that you would like to see covered in this educational content?

14

Where (which platforms/mediums) would you like to see this educational content around FASD diagnosis and clinical coding provided?

- ☐ Newsletter
- ☐ Social media
- ☐ Training course
- ☐ Physical information in workplace
- ☐ Other

15

In which format would you like to see this educational content around FASD diagnosis and clinical coding provided?

- ☐ Brief text summary
- ☐ Short video/animation (< 3 mins)
- ☐ Infographic/poster
- ☐ As part of training/educational resources (e.g. medical school/CPD courses)
- ☐ Other

Further contact:

16

Are you happy to be contacted regarding your responses, if necessary? *

If yes, please provide your contact details in the next three questions

◯ Yes

◯ No

17

Name (optional)

18

Email Address (optional)

19

Telephone (optional)

20

Would you like receive email updates on this project?

◯ Yes

◯ No

21

Would you be interested in being involved in a 1-hour online workshop to co-develop educational materials to improve coding and diagnosis of FASD?

- ◯ Yes
- ◯ No
- ◯ Maybe

22

Is there anything else you would like to tell us in relation to this project?
(please add any comments below)

This content is neither created nor endorsed by Microsoft. The data you submit will be sent to the form owner.

Microsoft Forms
