# Supplementary material for: Collaborative knowledge mobilisation to increase awareness and use of new SNOMED clinical terms for fetal alcohol spectrum disorder in England
Source: Front Public Health. 2026 Aug 31;14:1794809. doi: 10.3389/fpubh.2026.1794809 (PMC13572132; doi:10.3389/fpubh.2026.1794809)
Supplement: Supplementary file 2 [file Image_2.pdf]

Topic Guide for clinical coding of FASD workshop – 25th June 2024 at 1pm

Title slide (5 mins)

- Introductions
- Housekeeping
  - Consent for recording including reason why we will record this
  - Cameras on if possible
  - Nature of conversation, participant-led
  - If more comfortable, use the chat function and type in a response, JP to monitor chat

Background & workshop outline (5 mins)

- In addition to slides –
  - Grant for knowledge mobilisation and development of creative communications
  - Work is on-going to implement these codes in clinical systems (EMIS, SystmOne etc)
  - We want these materials to represent and be co-developed by clinicians
  - Value of this work to wider UK devolved nations who plan to implement SNOMED into primary care

Session 1 (10 mins) Prompts - Facilitators and barriers of clinically coding FASD

Roughly broken down into

A) What needs to have occurred (investigations, correspondence) for you to code FASD in clinical records?
B) Are there any situations where the above has occurred but you remain hesitant to code this?
C) What are the factors that would prevent you from coding FASD?
D) What can be done to improve coding of FASD?

Primary care – Themes/prompts

- Previous experience (presentation of condition, diagnostic pathways, coding)
- Stigma associated with diagnosis
- Lack of training around diagnosis of FASD
- Confidence in assessing PAE
- Confidence in documenting PAE
- Impact of secondary care written communication on coding
- System level difficulties – e.g. with SNOMED interface
- Understanding of importance of clinical coding

For secondary care **–**

- Terminology use and variation – ICD or NICE/SIGN terminology
- Commissioning of services to diagnose
- Building specialist knowledge and support in secondary care to support local primary care around diagnostic pathways

Session 2 (25 mins) – building the creative materials

Introduction –

- Frame budget constraints and that video will likely have to be ~ 1 minute in length
- Video indicated to be beneficial from our survey (most popular option) followed by infographics/text summaries.

General discussions

- If the goal is building diagnostic awareness of FASD and improving clinical coding of the condition, what do the key messages need to be?
- What key knowledge would be helpful for GPs to know?
    - Prevalence of condition?
    - Invisibility of FASD with non-sentinel features
    - Data gap
    - Diagnostic pathways
    - Signposting to specialist support
- How can this be supportive of clinicians? I.e. what is reasonable to ask General Practitioners to do?
- How can we frame this to give the patient perspective?
- What aspects of the video would help it have impact with its intended audience of clinicians?

How can we tie this all together – Beginning, Middle, End?

Other materials

- What would be key to include in an infographic?
- What needs to be the focus?
- For text summaries, what wider information may be helpful to clinicians?

Session 3 (10 minutes) – Platforms or methods of dissemination

How might we reach –

- General Practitioners
- Paediatricians
- Psychiatrists
- Wider MDT health professionals
